# Glucose Metabolic Dysfunction in Neurodegenerative Diseases—New Mechanistic Insights and the Potential of Hypoxia as a Prospective Therapy Targeting Metabolic Reprogramming

**DOI:** 10.3390/ijms22115887

**Published:** 2021-05-31

**Authors:** Rongrong Han, Jing Liang, Bing Zhou

**Affiliations:** 1 Beijing Advanced Innovation Center for Big Data-Based Precision Medicine, Interdisciplinary Innovation Institute of Medicine and Engineering, Beihang University, Beijing 100191, China; hanrongrong2019@buaa.edu.cn (R.H.); liangjing2019@buaa.edu.cn (J.L.); 2 School of Engineering Medicine, Beihang University, Beijing 100191, China

**Keywords:** brain energy metabolism, glucose, neurodegenerative disease, hypoxia, metabolic reprogramming

## Abstract

Glucose is the main circulating energy substrate for the adult brain. Owing to the high energy demand of nerve cells, glucose is actively oxidized to produce ATP and has a synergistic effect with mitochondria in metabolic pathways. The dysfunction of glucose metabolism inevitably disturbs the normal functioning of neurons, which is widely observed in neurodegenerative disease. Understanding the mechanisms of metabolic adaptation during disease progression has become a major focus of research, and interventions in these processes may relieve the neurons from degenerative stress. In this review, we highlight evidence of mitochondrial dysfunction, decreased glucose uptake, and diminished glucose metabolism in different neurodegeneration models such as Alzheimer’s disease (AD), Parkinson’s disease (PD), amyotrophic lateral sclerosis (ALS), and Huntington’s disease (HD). We also discuss how hypoxia, a metabolic reprogramming strategy linked to glucose metabolism in tumor cells and normal brain cells, and summarize the evidence for hypoxia as a putative therapy for general neurodegenerative disease.

## 1. Introduction

Although it only accounts for 2% of the body’s total weight, the brain has a high demand for energy compared to other tissues. Glucose is an essential energy substrate for the adult brain, and at least 25% of glucose is used to maintain basal brain functions [1]. Approximately 70% of the calculated energy is used for neuronal signaling (resting potentials, action potentials, postsynaptic receptors, the glutamate cycle, and postsynaptic Ca^2+^), and approximately 30% is used for non-signaling conduction activities (proteins, phospholipids, etc.). Glucose metabolism promotes the physiological functions of the brain through glycolysis and mitochondrial oxidative phosphorylation, and its product, ATP, is the electrochemical basis for the maintenance of neurons and non-neuronal cells [2]. Therefore, glucose metabolism and mitochondrial functions are essential for maintaining normal neuronal function.

Increasing studies have found that an abnormal glucose metabolism, including mitochondrial dysfunction, is related to the development of neurodegenerative diseases, such as Alzheimer’s disease (AD), Parkinson’s disease (PD), amyotrophic lateral sclerosis (ALS), and Huntington’s disease (HD), which makes it promising to find a solution to these changes, which could improve the quality of life and prolong the survival of patients with neurodegenerative diseases. As a physical stimulus, hypoxia has a potential role in metabolic reprogramming and even has a protective effect on diseases of mitochondrial dysfunction [3]. In this review, we analyzed the energy metabolism in neurodegenerative diseases and summarized the evidence for hypoxia as a potential treatment for metabolic reprogramming.

## 2. Energy Metabolism in the Brain

### 2.1. Glucose Metabolism in the Brain

**Figure 1:**
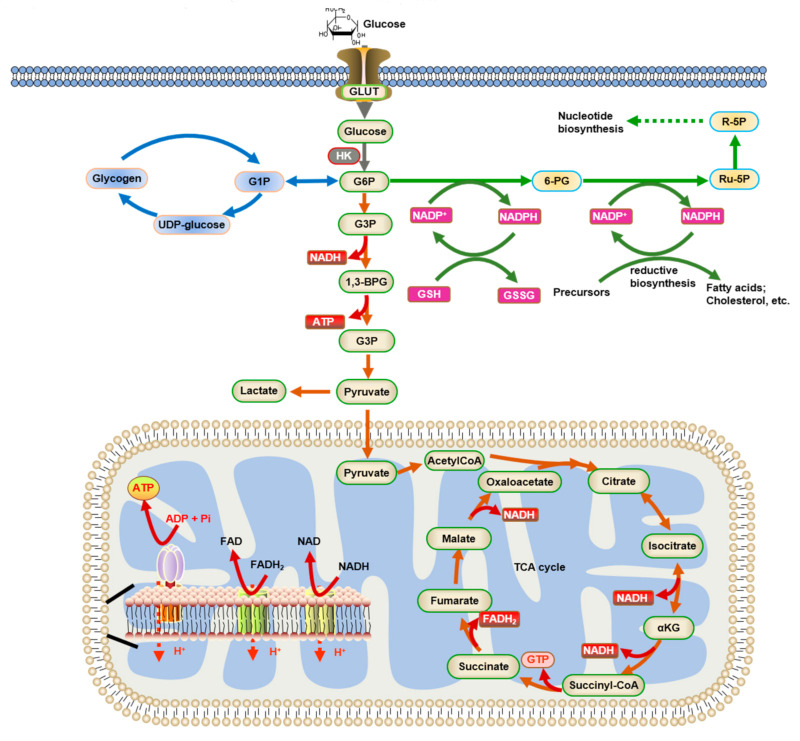
Glucose metabolism and energy homeostasis in the brain. Glucose enters the cell through GLUT and is converted to G6P by HK. Then, G6P can be processed by different metabolic pathways: (1) Glycolysis (shown by red arrows), which leads to lactic acid production or the tricarboxylic acid (TCA) cycle. NADH and FADH_2_ are subsequently re-oxidized in ETC to produce ATP. (2) The pentose phosphate pathway (PPP) (shown by green arrows), which metabolizes G6P and generates NADPH. NADPH is then used for oxidative stress defense and biosynthetic reactions. (3) Glycogenesis (shown by blue arrows). Abbreviations are as follows: GLUT: glucose transporters; HK: Hexokinase; G6P: glucose-6-phosphate; G1P: glucose-1-phosphate; 6-PG: 6-phosphogluconate; G3P: glyceraldehyde-3-phosphate; 1,3-BPG: 1,3-bisphosphoglycerate; Ru-5P: ribulose-5-phosphate; R-5P: ribose-5-phosphate.

In the brain, glucose is transported from the arterial blood across endothelial membranes into brain cells via specific glucose transporters, and this process is reversible. The glucose transporters are most commonly the GLUT transporter families, which have different kinetic properties, with GLUT1 located in endothelial cells and astrocytes and GLUT3 and GLUT4 located in neurons [4,5]. When glucose enters the cell, it is irreversibly phosphorylated by hexokinase (HK) to produce glucose 6-phosphate (G6P). Then G6P can be processed by different metabolic pathways: (1) glycolysis (leading to lactic acid production or the tricarboxylic acid (TCA) cycle), (2) the pentose phosphate pathway (PPP), and (3) glycogenesis (Figure 1).

Glycolysis is a non-oxygen-dependent metabolic pathway in which glucose is converted to two pyruvate molecules, resulting in the net production of two ATP, two H_2_O molecules, and two reduced nicotinamide dinucleotide (NADH) molecules [6]. The two pyruvate molecules resulting from glycolysis are then transported into the mitochondrial matrix and converted to acetyl-CoA via oxidative decarboxylation; thus, acetyl-CoA is complexed with citrate, which undergoes a series of regenerative enzymatic reactions (the TCA cycle). This transformation results in a total of two NADH molecules produced—one NADH per pyruvate molecule. Two TCA cycles produce a total of six NADH, two GTPs (similar to the ATP equivalent), two reduced flavin adenine dinucleotides (FADH2), and four CO_2_. The NADH produced during glycolysis and NADH and FADH2 produced during the TCA cycle is subsequently re-oxidized in the electron transport chain (ETC), which uses the energy generated by electron transfer through four major protein–metal complexes to transport protons across the inner mitochondrial membrane to the intermembrane space. The flux of protons back to the mitochondrial matrix is mediated by ATP synthase, which uses energy to produce ATP from ADP [7]. The coupling of the electron flow from NADH and FADH2 to molecular oxygen to the production of ATP represents a process known as oxidative phosphorylation. Oxidative phosphorylation produces ~32 ATP per glucose molecule oxidized. The complete oxidation of glucose produces larger amounts of energy in the form of ATP in the mitochondria compared to glycolysis. The metabolic fate of glucose in the brain depends on the cell type and the selective expression of metabolic enzymes. Neurons are predominantly oxidative, while astrocytes are predominantly glycolytic [8].

The PPP metabolizes G6P and generates NADPH, which is used for defense against oxidative stress and biosynthetic reactions. It also produces ribulose-5-phosphate that can convert to ribose-5-phosphate and is used in the biosynthesis of purine ribonucleotides or intermediates for other pathways. These intermediates rearrange to form fructose-6-phosphate plus G3P. In astrocytes, G6P can be produced from glucose and/or glycogen, while in neurons, it is derived only from glucose. The biosynthetic contribution of PPP is the greatest in the developing brain [9,10], whereas, in adults, NADPH production plays an important role in the glutathione (GSH) pathway. Glutathione hydrogen peroxide (together with catalase) is used to detoxify the superoxide produced by the respiratory chain and the biogenic amine neurotransmitters (serotonin, dopamine, and norepinephrine (NE)) metabolized by the monoamine oxidase and hydrogen peroxide that is produced [11]. In addition to inactivating other active compounds, such as methylglyoxal and formaldehyde [12], NADPH is also used in the biosynthesis of fatty acids and cholesterol and for nitric oxide synthase, cytochrome P450 reductase, aldehyde reductase, and aldose reductase, which can produce sorbitol from glucose under hyperglycemic conditions. The pentose phosphate shunt pathway has been historically considered to be a minor flux with a high capacity for upregulation across time [10].

Glycogen, a branched alpha-D glucose polymer, is the major fuel storage in the brain and is present at much lower concentrations than in other organs. In the presence of normal supplies of glucose, there are higher levels of glycogen in the brains of unstressed animals [13]. In the embryonic stages, glycogen appears in the glia and neuronal cells; however, in adults, glycogen is mainly found in astrocytes and is located throughout the cell: in the cytosol, the end-feet surrounding the vasculature, in perisynaptic processes, and is rarely detected in neurons [14]. More recently, small amounts of glycogen, glycogen synthase, and glycogen phosphorylase were detected in neurons using very sensitive assays [15]. Glycogenolysis is more efficient than glycolysis because the activation of glycogen phosphorylase is very fast, and glycogenolysis has almost a 50% higher ATP yield compared with the glycolytic metabolism of glucose. Three ATP are generated from glycogen-derived G6P versus two ATP from glucose-derived G6P because G6P is produced from glycogen without consuming ATP (the cost is “prepaid” during glycogen synthesis), except in the branched points where glucose is released by the debranched enzyme, and one ATP is consumed to produce G6P. Glycogen turnover is highly regulated to maintain normal functions [16], and its dysregulation with an abnormal accumulation of glycogen structures in the brain causes severe seizures and death in patients with Lafora disease [17].

### 2.2. Other Energetic Sources in the Brain

When glucose supply is restricted or insufficient, e.g., during prolonged fasting, the brain will utilize other substrates such as free fatty acids and amino acids for energy production in the form of ketone bodies. During fasting, free fatty acids are mobilized from adipocytes and transported to the liver, leading to a surplus production of acetyl-CoA, which does not enter the TCA cycle for ATP production but leads to the formation of ketone bodies [18]. Additionally, amino acids (particularly leucine) may contribute 4% of the total ketone body production in the post-absorptive state [19]. The major ketone bodies include acetoacetate (AcAc), acetone, and beta-hydroxybutyrate (BHB). The utilization of ketone bodies seems to depend mainly on the glucose concentration in the blood [20]. When ketone bodies in the plasma are raised either by fasting, diet, or infusion, they are transported to the brain by monocarboxylate transporters (MCTs), which are the only known transporters for ketone bodies and are distributed throughout the brain [21]. Neurons and astrocytes maintain the ability to absorb ketone bodies. Once BHB and acetoacetate are transported to the brain, they are converted back into acetyl-CoA, which enters the TCA cycle to produce ATP. Unlike glucose, the conversion of BHB and acetoacetate into an oxidizable form does not consume ATP [22]. In astrocytes, degradation of fatty acids results in ketone body release to neighboring neurons. Therefore, both exogenous and endogenous ketone bodies can provide fuel for the brain [23]. However, plasma ketone body levels are usually low after an overnight fast (<0.5 mM) and contribute to less than 5% of the brain’s metabolism. Notably, under normal physiological conditions, the brain primarily uses glucose to produce ATP [20].

**Figure 2:**
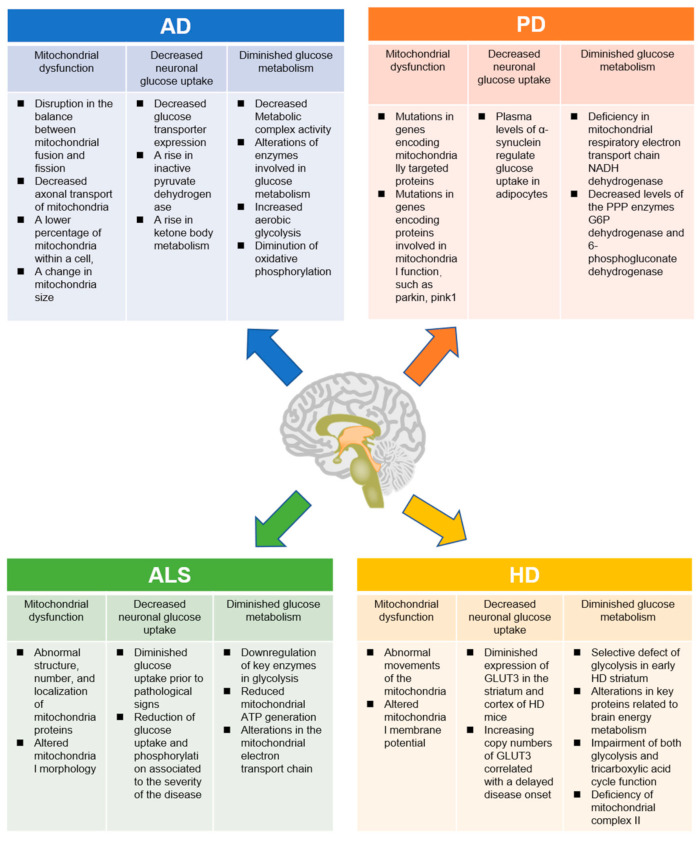
The crosstalk of metabolic dysfunction and neurodegenerative disease. Accumulating evidence has suggested the presence of a strong correlation between metabolic dysregulation and neurodegenerative disorders, such as AD, PD, ALS, and HD.

Approximately 90% of glucose is oxidized to CO_2_ to produce ATP through oxidative phosphorylation, which takes place in the mitochondria; therefore, mitochondria appear to be involved in the central stage of cellular energy supply, which is particularly important for the brain [24]. The metabolic intermediates of glucose are also used for the synthesis of amino acids for protein synthesis and neurotransmitter production, fatty acids and other lipids required for membrane and myelin synthesis, and five-carbon sugars required for nucleotide synthesis [8]. Thus, any disturbance in the neuronal glucose metabolism, primarily supported by the mitochondria, would affect neuronal function and ultimately affect movement, cognition, learning, and memory. Changes in the mitochondrial energy-transducing capacity, including a decreased neuronal glucose uptake, decreased ETC activity, and glucose metabolic disorders, can be detected in neurodegenerative diseases, including AD, PD, ALS, and HD (Figure 2).

## 3. Glucose Metabolic Disorders in Neurodegenerative Diseases

Neurodegenerative diseases are marked by the progressive loss of neuronal function, and thus far, there are very limited options for their treatment, which leaves these diseases associated with high morbidity and mortality. These different neurodegenerative diseases share two important characteristics: first, systemic loss of the neurons in the motor, sensory, and cognitive systems leading to cognitive disabilities, such as dementia and motor decline [25]; and second, a correlation between energy metabolic changes and neurodegeneration [26]. Given the limited regenerative ability of neuronal tissue, it is important to restrict neuronal impairment and death. The processes in neurodegenerative disease are quite complex. As deficits in glucose availability and mitochondrial function are well-known hallmarks of many neurodegenerative diseases, it appears reasonable to hypothesize that the high energy demand of the brain renders it sensitive to changes in the energy fuel supply and mitochondrial function. Therefore, it is of primary significance to interpret the neuronal damage in light of the metabolic changes and to look for therapies that can remedy the energy supply of the mitochondria.

### 3.1. Alzheimer’s Disease (AD)

AD was first described as “presenile dementia” by Alois Alzheimer in 1906 [27]. Alzheimer’s first AD patient was a 51-year-old female with a progressive loss of cognitive functions (comprehension and memory, unpredictable behavior, etc.). The histological analysis of her brain postmortem found senile plaques (SPs) and neurofibrillary tangles (NFTs), which are recognized as two main pathological findings in the brain of AD [28]. SPs are extracellular aggregates composed of amyloid β (Aβ) peptides, while NFTs are intracellular aggregates composed of hyperphosphorylated Tau protein [29,30]. AD begins with the memory loss of recent events (short-term memory impairment) and ultimately deprives the patients of self-awareness, and it is the most common form of dementia involving 50% to 70% of dementia cases. Nearly half of people over the age of 85 suffer from AD.

Some of the first clues hinting at energetic metabolism disorders in AD evolved from observations of the altered brain glucose metabolism in AD patients, which is detectable in living subjects using fluorodeoxyglucose positron emission tomography (FDG-PET) [31,32]. The hypometabolism of glucose and deficits in ATP production is observed in AD [33,34]. Hypometabolism is a key feature of AD that is manifested by mitochondrial dysfunction, decreased neuronal glucose uptake, and diminished glucose metabolism (before Aβ and tau tangles have begun forming) [24].

Extensive studies have indicated that mitochondrial structural dynamics are altered in both AD patients and animal models [35], including a disruption of the balance between mitochondrial fusion and fission, the decreased axonal transport of mitochondria, a lower percentage of mitochondria within a cell, and a change in size, with the mitochondria in AD maintaining much shorter, wider shapes [36]. Quantitative ultrastructural morphometric analysis showed that AD brains contained a significantly lower percentage of normal mitochondria compared with the age-matched control group brains [37] and a significantly higher percentage of mitochondria with broken cristae [38]. In fibroblasts from sporadic AD patients, the mitochondria were longer, with two or more mitochondria often joined together, while those of age-matched normal human fibroblasts were much shorter and appeared as sausage-shaped or rounded [39]. Similar morphological changes were also found in neurons overexpressing wild-type APP. APP-overexpressing cells actually showed mitochondria with heterogeneous morphologies; approximately 50% of cells contained fragmented, punctiform mitochondria, and the mitochondria in some cells demonstrated elongated, net-like structures [40].

Decreased expression of the glucose transporter has also been observed in AD. In a mouse model experiment, there was a decline in the neuronal GLUT3 both in male and female 3×TG-AD mice, which coincides with the brain glucose uptake. However, GLUT4 decreased in male 3×TG-AD mice but not in females [41]. Glucose uptake was also associated with a rise in inactive (phosphorylated) pyruvate dehydrogenase and in ketone body metabolism [42]. In an APP/PS1 model, compared to the wildtype, there was a decreased level of GLUT1 expression in the hippocampus of 18-month old mice, whereas no differences were observed at 8 months old [43]. Additionally, the GLUT1 and GLUT3 expression levels were reduced in six brain regions of AD patients, and the decreased GLUT3 levels in certain neurons compromised the glucose availability and may be responsible for the deficits in glucose metabolism [44]. Postmortem brains studies from AD patients revealed that the expressions of GLUT1 and GLUT3 were significantly decreased, which correlated with abnormal tau hyperphosphorylation and the downregulation of hypoxia-inducible factor 1α (HIF1α, which leads to the transcriptional activation of GLUT); interestingly, the GLUT2 expression was increased, likely due to astrocyte activation [45]. A large reduction in GLUT3 immunoreactivity was found in the dentate gyrus, a region where cells are selectively destroyed in AD [46]. Reduced GLUT expression in AD has also been found in the blood-brain barrier and cerebral cortex [47].

The activity of metabolic enzyme complexes in the TCA cycle decreased in AD, particularly for the pyruvate dehydrogenase complex (PDHC), the α-ketoglutarate dehydrogenase complex (KDHC), and enzymes in the ETC, such as succinate dehydrogenase (in complex II) and cytochrome c oxidase (COX, in complex IV) [48,49]. In 1990, deficient COX activity was found in the platelets of AD patients. A similar finding was made in AD brains in 1992 [50,51]. Subsequently, the finding of reduced COX activity in AD patients has been replicated in platelets [52,53,54], fibroblasts [55], focal brain regions [53], and large parts of the brain [56,57]. COX reduction has also been reported at all stages of the disease, including mild cognitive impairment [49,58]. In a 3xTG-AD model, there was a decreased expression and activity of complex IV in female mice coupled with compromised oxidative phosphorylation [59]. The dysfunction of mitochondrial complexes was also observed in clinical studies [60,61]. A proteomic study in AD patients found that the complex III core protein-1 in the temporal cortex and the complex V β-chain in the frontal cortex were significantly reduced [62], and, in another study, mitochondria from AD patients exhibited suppressed activity in all ETC complexes, with a dramatic decline in complex IV activity [51,63]. Additionally, research demonstrated that these deficits are not specific to AD but are also common across other neurodegenerative disorders [64].

Alterations of the enzymes involved in glucose metabolism were also observed in AD. The downregulation of the canonical WNT/β-catenin pathway inhibits β-catenin target genes (PDK1, MCT-1, c-Myc, cyclin D1, and LDHA) that participate in the regulation of glucose metabolism [65,66,67]. The downregulation of β-catenin also reduces the expression of the PI3K/Akt pathway [68,69]. HIF-1α, a downstream target of the PI3K/Akt pathway, is responsible for the expression of enzymatic enzymes, such as LDHA, PDK1, and PKM2 [70,71]. The levels of HK were found to be decreased in amyloidogenic AD transgenic mouse models as well in post-mortem brain tissues from AD patients [72]. Aβ-toxicity-resistant nerve cells undergo metabolic reprogramming and shifts toward aerobic glycolysis through the activation of HIF-1α, PDK1, and LDHA [73,74]. PDK1 and LDHA activation promotes resistance to Aβ toxicity and leads to the diminution of oxidative phosphorylation [75]. The acute treatment of Aβ induced microglia activation is accompanied by metabolic reprogramming from oxidative phosphorylation (OXPHOS) to glycolysis [76].

### 3.2. Parkinson’s Disease (PD)

PD is cited to be the second most common age-related neuronal degenerative disorder after AD and affects 0.6% of the population who are 65–69 years old and 2.6% of the population between 85–89 years of age [77]. The cardinal clinical features of PD are rest tremors, slowness of movement, rigidity, and postural instability [78]. In addition to motor symptoms, PD patients also show some non-motor symptoms, such as hyposmia/anosmia, sleep abnormalities, gastrointestinal motility disturbances, anxiety, depression, dementia, and impaired cognition [79]. The pathological hallmarks of PD are the progressive loss of dopaminergic neurons within the substantia nigra pars compacta (SNpc). Motor symptoms of PD do not become clinically apparent until 60–80% loss of dopaminergic neurons from the striatum, but many nonmotor symptoms may precede the onset of motor symptoms by years or decades [79]. The formation of Lewy bodies (LBs), which are composed primarily of post-translationally modified (ubiquitinated, phosphorylated, and/or S-nitrosylated) α-synuclein [80,81], and gliosis have been identified. In addition to the involvement of the nigrostriatal pathway, neurodegeneration and LBs are also found in the locus ceruleus, nucleus basalis, hypothalamus, cerebral cortex, cranial nerve motor nuclei, and central and peripheral components of the autonomic nervous system [79,82]. Large amounts of studies have demonstrated the mechanism and a variety of hypotheses have been proposed; however, the detailed mechanisms of PD remain unclear.

The idea that mitochondrial dysfunction could be implicated in the pathogenesis of PD was originally reported in the early 1980s by Dr. William Langston and colleagues. They found that a group of young drug abusers exposed to 1-methyl-4-phenyl-1,2,3,4-tetrahydropyridine (MPTP) displayed motoric features that bore an uncanny resemblance to those exhibited by sporadic PD patients [83]. It turned out that the active metabolite of MPTP results in marked dopaminergic neurotoxicity selectively for dopamine transporter and significant inhibition of mitochondrial complex I once it enters the neurons, which highlighted, for the first time, that mitochondrial dysfunction may be responsible for the neurodegeneration in PD. Among a number of proposed mechanisms involved in PD pathogenesis, mitochondrial dysfunction has been repeatedly implicated as the cause of the death of dopaminergic neurons in PD [84,85,86,87]. A number of familial forms of PD are associated with mutations in genes encoding both mitochondrially targeted proteins and proteins involved in mitochondrial function and/or oxidative stress responses. Examples of such genes are α-synuclein, Parkin, phosphate, and tensin homolog-induced kinase 1 (PINK1), DJ1, leucine-rich repeat kinase 2 (LRRK2), and Htr A serine peptidase 2 (HTRA2) [88]. Genetically modified organisms based on the knockout, overexpression or mutant versions of these genes have since been generated for the purposes of PD animal modeling. Many of these nuclear genes also implicate a role for the mitochondria in PD pathogenesis. α–synuclein can directly affect the mitochondria in mice that overexpress mutant α-synuclein [89]. In transgenic mice overexpressing α-synuclein, mitochondrial function is impaired, and oxidative stress increases [90]. Parkin, a ubiquitin ligase, is believed to protect neuron mitochondria, and researchers reported, in drosophila and mouse models, that parkin deficiency or mutations led to increased oxidative stress and mitochondrial impairment [91,92]. In contrast, mitochondrial dysfunction and oxidative stress also affect parkin function and exacerbate the consequences of parkin mutations [93]. PINK1, a mitochondrion-localized kinase, appears to protect against cell death [94]. This protective effect is abrogated by PD-related mutations that disable its kinase function [95]. PINK deficiency increases the sensitivity of mitochondria to rotenone and induces the degeneration of dopaminergic neurons in drosophila [96]. Consistent with the evidence from basic science, clinical studies also showed that mitochondrial damage plays a predominant role in the development of PD in patients.

Mitochondrial dysfunction could lead to metabolic disorders. Glucose hypometabolism in PD brains has also been documented using magnetic resonance imaging (MRI) and PET methods [97]. Studies demonstrated that the plasma levels of a-synuclein regulated glucose uptake in adipocytes [98]. The glycolytic enzyme glucose-6-phosphate isomerase, which catalyzes the conversion of G6P to F6P, was recently identified as a conserved modifier of dopamine metabolism, protein aggregation, and neurodegeneration in *Caenorhabditis elegans*, *Drosophila melanogaster*, and murine neurons [99]. Decreased levels of the PPP enzymes G6P dehydrogenase and 6-phosphogluconate dehydrogenase were detected at early stages in the putamen and cerebellum of PD brains [100]. A mild deficiency in mitochondrial respiratory electron transport chain NADH dehydrogenase (Complex I) activity has been reported in the substantia nigra [101], as well as in platelets [102,103] and lymphocytes [104,105] in PD patients, which suggests a systemic inhibition of complex I activity in PD patients. Interventions directed to improve mitochondrial bioenergetics have been shown to ameliorate neuropathology and motor deficits in animal models of PD [106].

### 3.3. Amyotrophic Lateral Sclerosis (ALS)

ALS is a progressive paralytic disease that was first clinicopathologically described by Jean-Martin Charcot in Paris in the mid-nineteenth century [107] and is characterized by the selective degeneration and death of motor neurons. ALS is the third most frequent neurodegenerative disease after AD and PD, of which the annual population incidence is 2/100,000 per year. ALS is categorized in two forms. The most common form is sporadic (90–95%), which has no obvious genetically inherited component. The remaining 5–10% of the cases are familial-type ALS due to their associated genetic dominant inheritance factor [108,109,110,111,112,113]. The first onset of symptoms is typically between the ages of 50 and 65 [114,115,116,117]. The most common symptoms that appear in both types of ALS are muscle weakness, twitching, and cramping, which eventually can lead to the impairment of muscles [118,119]. In the most advanced stages, ALS patients will develop symptoms of dyspnea and dysphagia [120,121]. The malignant nature of the disease progression is indicated by the fact that the median survival is approximately 2 years from diagnosis and 3 years from symptom onset [122]. However, there is considerable clinical variation, with a long ‘tail’ in the survival curve; approximately 10% of patients are still alive 10 years after onset. A better understanding of the biological underpinning of this variation in outcomes could shed considerable light on the nature of ALS.

Abnormal structures, numbers, and localizations of mitochondria have been reported in the motor neurons, muscles, and intra-muscular nerves of ALS patients [123,124,125]. Altered mitochondrial morphology in motor neurons is also one of the earliest pathological signs in mutant SOD1 mouse models of ALS [126,127,128]. Mutant SOD1 mice show vacuolated mitochondria in motor neurons that are lacking any apoptotic features [128,129]. In addition, mitochondrial morphology is also abnormal in TDP-43 models [130,131].

Mitochondrial abnormalities may lead to defects in glucose utilization. Studies have shown that the glucose uptake and levels within CNS tissues were severely diminished prior to pathological signs in SOD1^G93A^ mice [132,133], and abnormalities in glucose utilization in the cerebral motor cortex were accompanied by marked reductions in ATP generation; however, glucose utilization in the spinal cord was unaltered at this stage but became impaired with disease progression [132]. Decreased glucose uptake was also seen in the motor-sensory cortex of ALS patients [134,135]. One FDG-PET study consisting of 81 patients with a suspected diagnosis of ALS was able to correctly classify 95% of ALS cases [136]. Another FDG-PET study showed that a reduction in glucose uptake and phosphorylation was associated with the severity of the disease [137]. Glucose uptake in the spinal cord increased pre-symptomatically in mutant SOD1^G93A^ mice but declined progressively during disease progression [133]. These studies indicate that reduced glucose uptake may be an early diagnostic event in ALS.

Diminished glucose metabolism has been repeatedly reported in numerous studies. The expression of phosphoglucomutase-2-like 1 (PGM2L1) and phosphoglycerate kinase (PGK), two key enzymes in glycolysis, is downregulated in fibroblasts from sporadic ALS patients, which may lead to the reduction in the ability to catabolize carbohydrates in human ALS patients [138]. In agreement, a marked reduction in the components of glycolysis was observed in a recent proteomic study in sporadic ALS skin fibroblasts [139]. Whole-genome expression profiling in the motor cortex of sporadic ALS patients also showed significant downregulation of glycolytic genes [140]. Another study in the post-mortem cortex of ALS patients identified an over two-fold reduction in the PFKFB3 mRNA content [141]. In contrast, there are also studies showing that ALS patients displayed increased systemic energy expenditure at rest and were hypercatabolic [142]. Introducing mutant SOD1 in human fibroblasts or NSC34 motor neuron-like cells was found to increase glycolysis and reduce mitochondrial ATP generation [143,144]. In line with this, a recent metabolomics analysis identified increased glycolysis and deficits in the amino acid metabolism in a cellular model of ALS. Researchers have hypothesized that the overall increase in energy demands may reflect major cellular activity to stimulate CNS and muscle repair in an attempt to control the ALS neurodegenerative process [145]. Given the limited capacity of neurons to upregulate glycolysis [146,147], the physiological relevance to ALS regarding the upregulation of glycolysis in these cells remains to be established. Nevertheless, neurons were found to upregulate glycolysis [148], and oxidative stress was evident in post-mortem samples of ALS patients [149]. The inconsistency in the studies above may reflect different metabolic changes in different disease stages. Additionally, many studies have reported alterations in the mitochondrial electron transport chain, such as reduced complex I activity and cytochrome c oxidase activity in the skeletal muscle, spinal cords, and motor cortex of ALS patients [150,151,152,153]. The maximal oxidative phosphorylation capacity of skeletal muscle mitochondria was significantly increased in early-stage ALS patients, and the muscular mitochondrial respiratory complex IV activity was significantly decreased as the disease advanced [154]. Impaired activities of the complexes I + III, II + III, and IV were also observed in mutant SOD1^G93A^ mice [155]. In SOD1^G93A^ mice, decreased activity of mitochondrial complex I was detected as early as 2 months [155,156]. Together, these findings indicate that altered metabolic homeostasis is associated with ALS pathology and varies according to the disease progression.

### 3.4. Huntington’s Disease (HD)

HD was first described by George Huntington in 1872. This is an autosomal-dominant inherited disease that affects approximately 5–10 individuals out of 100,000 worldwide [157]. The clinical syndromes are characterized by abnormal choreic involuntary movements, psychiatric, psychological, and intellectual disabilities, and radiologically characterized by varying degrees of striatal atrophy [158]. HD is caused by an expansion of the CAG tract within exon 1 of the huntingtin gene (htt), which encodes a polyglutamine stretch in the HTT protein. In affected individuals, the number of CAG repeats extends from the normal population range (an average of 16 to 20 repeats) to >35 replicates [159], with a maximum expansion of 121 trinucleotides observed [160]. Glutamine itself is not toxic as it is present in all humans. However, the expansion of the polyglutamine tract is prone to misfolding and the formation of toxic aggregates, which may be one of the factors leading to HD [161,162]. This aggregation is responsible for secondary complications, such as apoptosis, excitotoxicity, mitochondrial dysfunction, and transcriptional disorders, which lead to the progressive loss of neurons in the brain, specifically in the striatum and cortex, and ultimately lead to disturbed neuropathological features.

Mitochondrial morphology has been widely observed to be impaired in HD. *htt* controls both the anterograde (from the cell body to axon terminal) and retrograde (from the axon ends toward the cell body) movements of the mitochondria [163]. Normal *htt* interacts with *htt*-interacting proteins 1 and 14 (Hip1 and Hip14), endophilin3, clathrin, and dynamin to control the fission and fusion processes of mitochondria [164]. Normal huntingtin localizes in the mitochondrial outer membrane, which makes the mitochondria vulnerable to any mutations of *htt* [165]. *htt* also has a role in the regulation of the mitochondrial membrane potential [166]. Thus, the functions of the mitochondria could certainly be affected in HD.

Energy metabolic deficits were also reported in numerous studies. Studies of the cerebral glucose metabolism using F-18 fluorodeoxyglucose PET provide strong evidence for an impairment of the energy metabolism in the caudate putamen and cortex of presymptomatic HD patients [167]. The striatal metabolism has been shown to be decreased prior to atrophy, and the disease progression is strongly correlated with glucose hypometabolism [168]. In line with this, research has observed that during the early stages of striatum degeneration, HD patients displayed decreased brain glucose uptake [169]. The expression of GLUT3 was shown to be diminished in the striata and cortices of HD mice compared to wild-type mice [170]. Interestingly, increasing the copy numbers of the gene encoding GLUT3 correlated with delayed disease onset in HD patients, and the overexpression of GLUT3, phosphofructokinase, and G6P-dehydrogenase protected against the development of HD phenotypes in animal models [171].

The study of the mitochondrial oxidative metabolism in the striatum of presymptomatic HD patients with PET through directly measuring the molar ratio of the cerebral oxygen metabolism to the cerebral glucose metabolism demonstrated a selective defect of glycolysis in early HD striatum [172]; these data suggest that a metabolic deficit is an early event in HD, and metabolic impairment precedes neuropathology and clinical symptoms in HD patients. Another study showed that an impaired basal ganglia metabolism was highly correlated with the functional capacity of HD patients and the degree of their motor dysfunction [173]. A system-wide analysis of the spatial proteome combined with mass spectrometric analysis recently identified alterations in key proteins related to the brain energy metabolism, and particularly the glia metabolism in a mouse model of HD [174]. In HD patients, increased lactate levels were observed in the striatum and occipital cortex using magnetic resonance spectroscopy imaging [175], which may reflect inefficient oxidative phosphorylation, which leads to the accumulation of lactate from pyruvate via lactate dehydrogenase. However, another magnetic resonance spectroscopy study showed reduced levels of both lactate and citrate in the cerebrospinal fluid from HD patients, which may indicate impairment of both glycolysis and the tricarboxylic acid cycle function in HD patients [176]. Enzyme complex abnormalities were also seen in HD. Postmortem studies showed a marked deficiency of mitochondrial complex II in the striatum of HD patients [177,178]. Decreased complex II enzymatic activity associated with the selective depletion of succinate dehydrogenase (SDH) was observed in cultured striatal neurons transfected with N-terminus mutant *htt*, and the overexpression of complex II/SDH subunits had a protective effect in this model [179]. The expression of full-length mutant *htt* in immortalized striatal progenitor cells (derived from the HdhQ111 knock-in mouse model) decreased complex II activity and increased the sensitivity of cells to a Ca^2+^-induced decrease in oxygen consumption and the mitochondrial membrane potential, whereas the overexpression of complex II prevented mitochondrial dysfunction and cell death [180]. These studies indicate that interventions that improve neuronal energy metabolism may ameliorate HD pathogenesis.

In summary, energy metabolic disorders caused by mitochondrial dysfunction play an important role in the development of neurodegenerative diseases. As hypometabolism is a hallmark of neurodegenerative diseases and the mitochondria are the main supplier of cellular energy, mitochondrial dysfunction is bound to influence the energy metabolism in a cell. Therefore, we speculate that strategies that can alter the glucose metabolism pathway to compensate for the metabolic disorder caused by mitochondrial dysfunction may have a therapeutic effect on neurodegenerative diseases. Here, hypoxia may be a potential strategy.

## 4. Hypoxia and Glucose Metabolic Reprogramming

### 4.1. Hypoxia Response In Vivo

**Figure 3:**
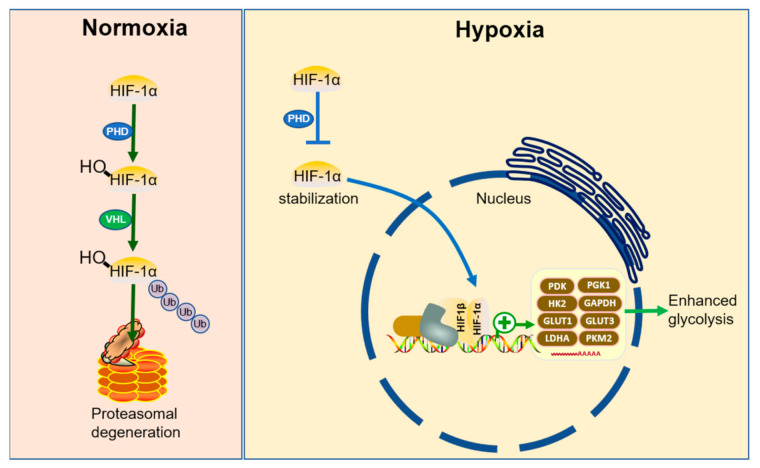
Glucose metabolic reprogramming induced by hypoxia. In normoxia conditions, HIF is constitutively made and hydroxylated by prolyl-hydroxylase (PHD) enzymes; the hydroxylated form is recognized by ubiquitin ligase, Von Hippel-Lindau (VHL), and targeted for proteasomal degradation. In response to the environmental hypoxia, the PHD reaction does not take place, allowing HIF stabilization and translocation to the nucleus. HIF binds with other transcriptional factors to enhance the transcription of genes encoding the enzymes involved in glycolysis.

Hypoxia (a low oxygen availability) in vivo can be caused by not only an insufficient oxygen supply from the local circulatory system (e.g., in cancer, ischemia in the heart or brain, and in embryos), but also increased oxygen consumption by cells engaged in certain functions (e.g., inflammation, proliferation, and hormone secretion). Hypoxia-inducible factor (HIF) is an important transcription factor that regulates oxygen consumption and morphologically changes in response to hypoxic stress in normal and pathological conditions by activating the transcription of numerous genes responsible for oxygen delivery, angiogenesis, cell proliferation, cell differentiation, and metabolism [181,182]. In normoxic conditions, HIF is constitutively synthesized and hydroxylated by the prolyl-hydroxylase (PHD) enzymes [183,184,185]. The hydroxylated form is recognized by the ubiquitin ligase Von Hippel-Lindau (VHL) and targeted for proteasomal degradation. In hypoxic conditions, the PHD reaction does not take place, allowing HIF stabilization and activation of the hypoxia transcriptional program (Figure 3).

In vivo, cells have developed two methods of dramatic alterations in the energy metabolism to adapt to oxygen deprivation: one is oxygen-independent ATP production, and the other is a reduction in the mitochondrial oxygen consumption. The former is the enhancement of glycolysis. The expression of glucose transporters (GLUT1 and GLUT3) [186,187] and glycolytic enzymes, including hexokinase (HK1 and HK2) [188] and phosphoglycerate kinase 1 (PGK1), are activated by HIF, which is stable in hypoxia conditions [189]. In addition, HIF also upregulates lactate dehydrogenase A (LDHA), which regenerates NAD+ for a continuous supply in glycolysis [190]. Although glycolysis produces less ATP per glucose molecule than oxidative phosphorylation, the cooperative induction of glucose uptake and glycolysis can result in rapid energy production that compensates for the low efficiency. The second is metabolic suppression in mitochondria to decrease oxygen consumption. The activity of ETC complexes is also suppressed. The hypoxic alterations in the energy metabolism are essential for the functions of different cells; for example, hematopoietic stem cells can maintain their stemness in the bone marrow niche, where the oxygen concentrations are low enough to activate HIF constitutively under physiological conditions [191,192].

### 4.2. Hypoxia and Tumor Metabolic Reprogramming

Normal tissue uses glycolysis to generate approximately 10% of the cell’s ATP, with mitochondria accounting for 90%. In contrast to normal cells, cancerous cells have long been known to show high glycolytic rates even under normoxic conditions (Warburg effect) [193], and over 50% of the cellular energy is produced by glycolysis, with the remainder being generated in the mitochondria. This shift is termed aerobic glycolysis, as it occurs even when there is enough O_2_ present to support mitochondrial functions. Tumor cells consume more glucose during glycolysis for energy production because of the low efficiency of glycolysis in generating ATP [194].

One of the most recognized reasons for the altered tumor metabolism is the unique physiological stresses that exist within the tumor. The tumor microenvironment suffers from hypoxia, acidosis, and increased interstitial fluid pressure [195]. These microenvironmental stresses are largely the result of poorly formed tumor vasculature [196]. Hypoxia is perhaps the most pervasive of these stresses and variably exists when O_2_ delivery does not meet the demands within the tumor tissue. Tumor cells respond to hypoxia conditions and adapt their metabolism to adjust the O_2_ demands to meet the limited supply [197,198]. Perhaps the most important aspect of how cells respond to this unique microenvironment is the activation of HIF-1 [199]. The net result of hypoxic HIF-1 activation is to shift the energy production by increasing glycolysis and suppressing the mitochondrial function. HIF-1 promotes glycolytic energy production by activating the transcription of genes involved in extracellular glucose import (such as GLUT1) and the enzymes responsible for the glycolytic breakdown of intracellular glucose (such as phosphofructokinase 1 (PFK1) and aldolase). Additionally, HIF-1 also downregulates oxidative phosphorylation within the mitochondria by transactivating genes, such as pyruvate dehydrogenase kinase 1 (PDK1) [200,201] and MAX interactor 1 (MXI1) [202]. It has been shown that oncogenes, such as c-Myc and v-Src promote metabolic reprogramming, in part, by cooperation with and activation of HIF-1, respectively [203,204]. In addition, the loss of tumor suppressor genes, including PTEN [205] and VHL [206], is involved in the development of Warburg effects via HIF-1 activation, independent of oxygen concentration.

Not limited to glucose, tumor cells also develop additional nutrition sources to bypass oxygen limitations. Studies reported that tumor cells consume acetate and ketone bodies to fuel tumor cell growth and metastases [207,208,209]. Hypoxia-produced lactate also serves as a carbon source for tumor cells. In human non-small-cell lung cancers, lactate was absorbed avidly by MCT1 and subsequently transformed into pyruvate to fuel the TCA cycle and facilitate cancer progression [210,211]. A recent study performed genome-wide CRISPR growth screens at 21%, 5%, and 1% oxygen to systematically identify gene knockouts with relative fitness defects in high oxygen or low oxygen. The knockouts of many mitochondrial pathways thought to be essential, including complex I and enzymes in Fe-S biosynthesis, grew relatively well with low oxygen, indicating that hypoxia could buffer mitochondrial defects. By contrast, in certain cell types, the knockout of lipid biosynthetic and peroxisomal genes caused fitness defects only with low oxygen [212]. These data suggest that hypoxia may promote metabolic reprogramming by modulating mitochondrial functions.

### 4.3. Hypoxia and Brain Metabolic Reprogramming

Though the severe hypoxic condition caused by ischemic stroke triggers neuronal cell death, mild oxygen deprivation or low oxygen could promote cortical progenitor cell expansion [213] and neurogenesis [214], suggesting a regulatory mechanism of oxygen tension in the brain. Consistently, in response to hypoxia, the brain also experiences a series of gene expressions such as HIF and its targeting genes and glucose metabolic changes. As in other normal cells discussed above, brain cells also temper their reliance on oxidative phosphorylation and concomitantly upregulate glycolytic enzymes and glucose transporters to promote glycolysis, which makes glycolysis a more prominent role in cellular ATP production to meet cellular energy requirements [215]. Notably, both ATP demand and supply downregulation are essential mechanisms of hypoxia tolerance [216]. The resulting attenuated electron flux through the mitochondrial respiratory chain has been demonstrated to be at least partially due to inhibition of complex I activity [217]. Compare with astrocytes that are highly glycolytic, neurons rely on oxidative metabolism to meet their high energy needs and are more sensitive to hypoxia. As the energy metabolic complement at the cell level, the astrocyte-neuron lactate shuttle is known as the way for astrocytes to support the energy expenditure of neurons [8]. Controversially, though, the neuron could consume glucose directly in response to the energy expenditure challenges [148], which mimics energy metabolism regulation and reprogramming during hypoxia.

### 4.4. Hypoxia and Neurodegenerative Disease

Metabolic reprogramming with hypoxia is critical for aerobic cells and organisms to survive as the oxygen concentrations surrounding them vary drastically in disease conditions and even under physiological ones. Jain et al. published in 2016 that hypoxia had protective effects on mitochondrial disease. They found that the hypoxia response elicited by genetic or small molecule activation had a protective effect on mitochondrial toxicity in cultured cells and zebrafish models. Specifically, activation of HIF through VHL knockout or FG-4592 treatment alleviated zebrafish embryo death caused by mitochondria respiratory chain inhibition. Furthermore, chronic hypoxia treatment benefited the genetic mouse model of Leigh syndrome, the most common pediatric manifestation of mitochondrial disease, and the survival rates, behavior, neuropathology, body weight, and disease biomarkers were significantly improved [3]. In 2017, one study also reported that breathing normobaric 11% O_2_ prevents neurodegeneration in Leigh syndrome, and hypoxia can prevent and even reverse the brain lesions in mice with advanced neuropathology [218]. Notably, when grown in 1% ambient O_2_, FXN-null yeast, human cells, and nematodes were fully viable. In human cells, hypoxia restored the steady-state levels of Fe-S clusters and normalized the ATF4, NRF2, and IRP2 signaling events associated with Friedreich’s ataxia (FRDA). In the mouse model of FRDA, breathing 11% O_2_ can significantly reduce the progression of ataxia [219]. In addition, intermittent hypoxic conditioning (IHC) prevented anxiety-like behavior and memory and learning deficits and significantly reduced cortical Aβ levels in 3×Tg-AD mice. In affecting the brain energy metabolism, IHC caused a significant increase in brain cortical glucose levels and robustly improved mitochondrial bioenergetic profile in 3×Tg-AD mice [220]. Another pilot clinical study examined the effect of intermittent hypoxia-hyperoxia training (IHHT) in elderly patients with mild cognitive impairment (MCI), the precursor to AD. IHHT significantly promoted cognitive function and functional exercise capacity in these elderly patients who received a multimodal training intervention [221]. Consistent with this, moderate IHC adaptation can improve cerebral oxygenation and hypoxia-induced cerebral vasodilation in elderly patients with MCI while improving short-term memory and attention [222]. IHHT also improved cognitive test scores and decreased the Aβ expression and NET formation significantly one month after the three-week IHHT intervention [223], suggesting a long-term neuroprotective mechanism for neurodegeneration disease of hypoxia.

In addition, high-altitude hypoxia may also have neuroprotective effects. Richardson et al. reported that there were no adverse effects of chronic hypoxia in adolescents who resided from birth at 3700 m in Bolivia, and instead, evidence of successful neurophysiological adaptations was found [224]. Another study showed that in California, where the average altitude is as high as 1800 m, Alzheimer’s disease mortality was negatively correlated with residential altitude [225]. Populations in certain high-altitude may also have special protection against dementia, such as a tribe in the Himalayas of North Indian [226]. Recently, it has been shown that IHC (simulation at 5000 m height, and 4 h per day for 15 consecutive days) can reduce cognitive deficits and anxiety in 9 month-old APP/PS1 mice while reducing amyloid β and pro-apoptotic protein contents in the cerebral cortex and hippocampus and augmenting hippocampal neurogenesis and BDNF content [227]. Together, these studies suggested that hypoxia has potential use as a new nonpharmacological therapy to improve the cognitive function of patients with neurodegenerative diseases and slow the development of neurodegenerative diseases.

## 5. Conclusions and Future Perspectives

Abnormal energy metabolism and mitochondrial dysfunction appear to have a role in neurodegenerative disease. Mitochondrial dysfunction dramatically decreases the energy supply to the neurons, which may aggravate the progress of neurodegeneration. As a consequence, targeting defects in the energy metabolism in neurodegeneration represents a rational therapeutic strategy. A strategy that manipulates the energy metabolism would be a particularly potent therapy to treat neurodegenerative disease. Hypoxia, as a treatment strategy, activates an evolutionarily conserved adaptive program that allows mammals to cope with limiting oxygen levels, and this program decreases an organism’s reliance on mitochondrial oxidative metabolism. This metabolic alteration may be helpful to improve neurodegenerative disease. Further preclinical studies are required to assess whether hypoxic exposure can be developed into a safe and effective treatment for human diseases associated with mitochondrial dysfunction.

## Data Availability

Not applicable.
